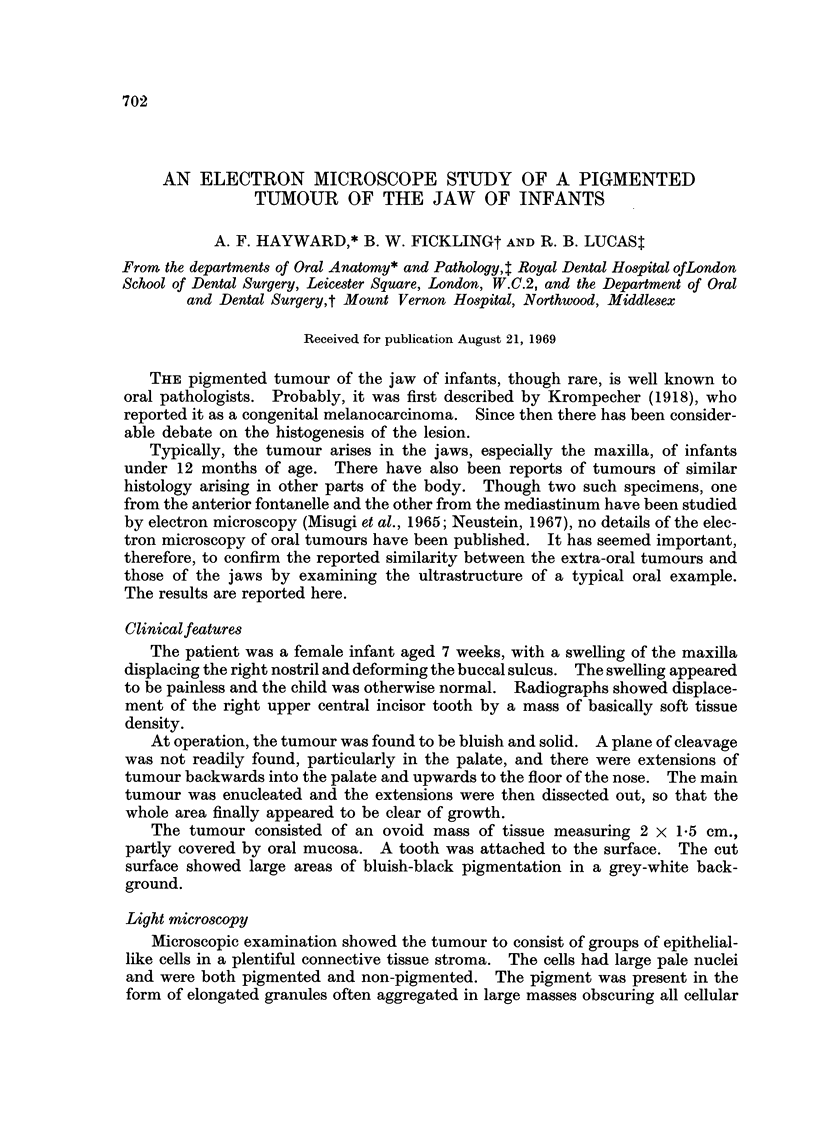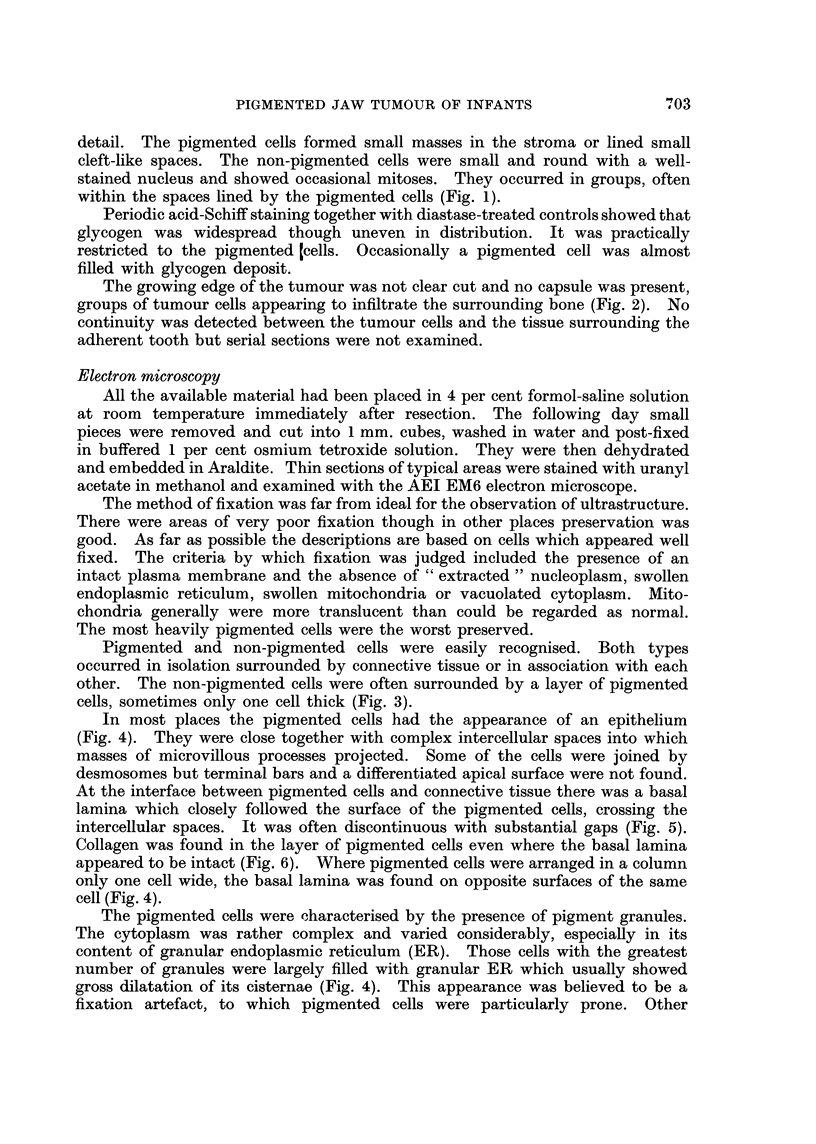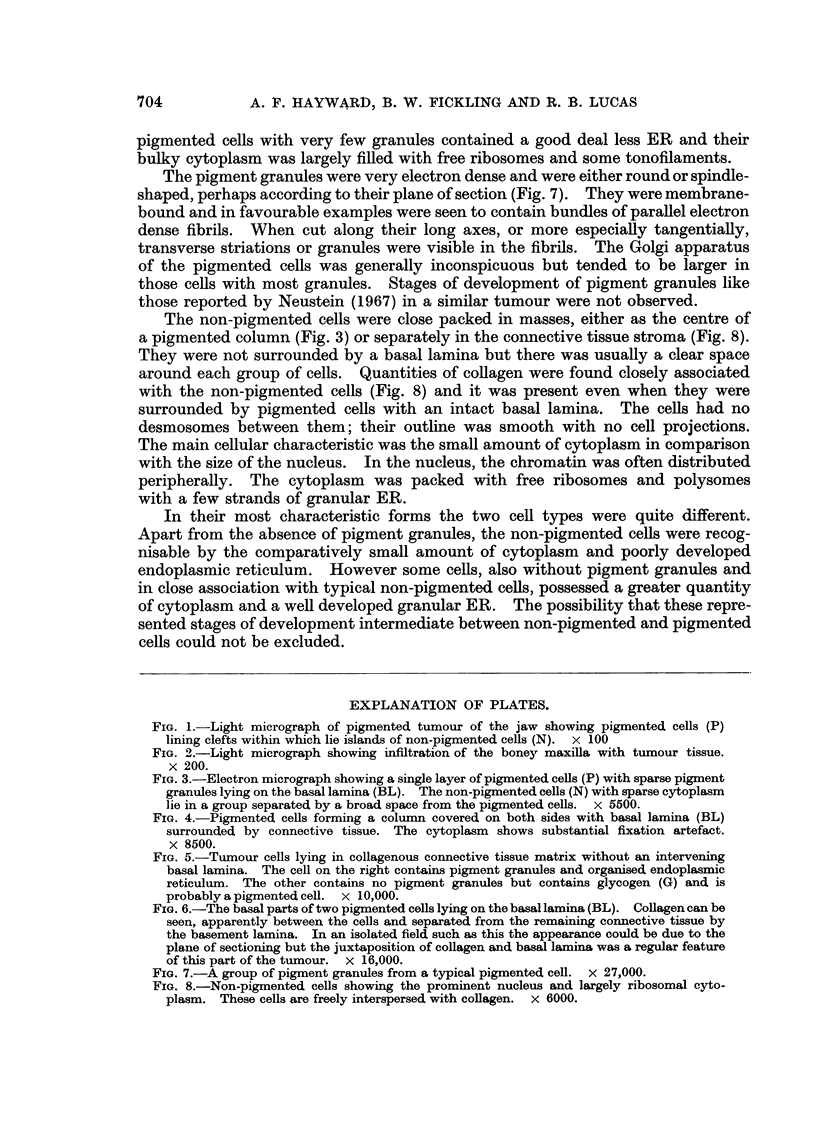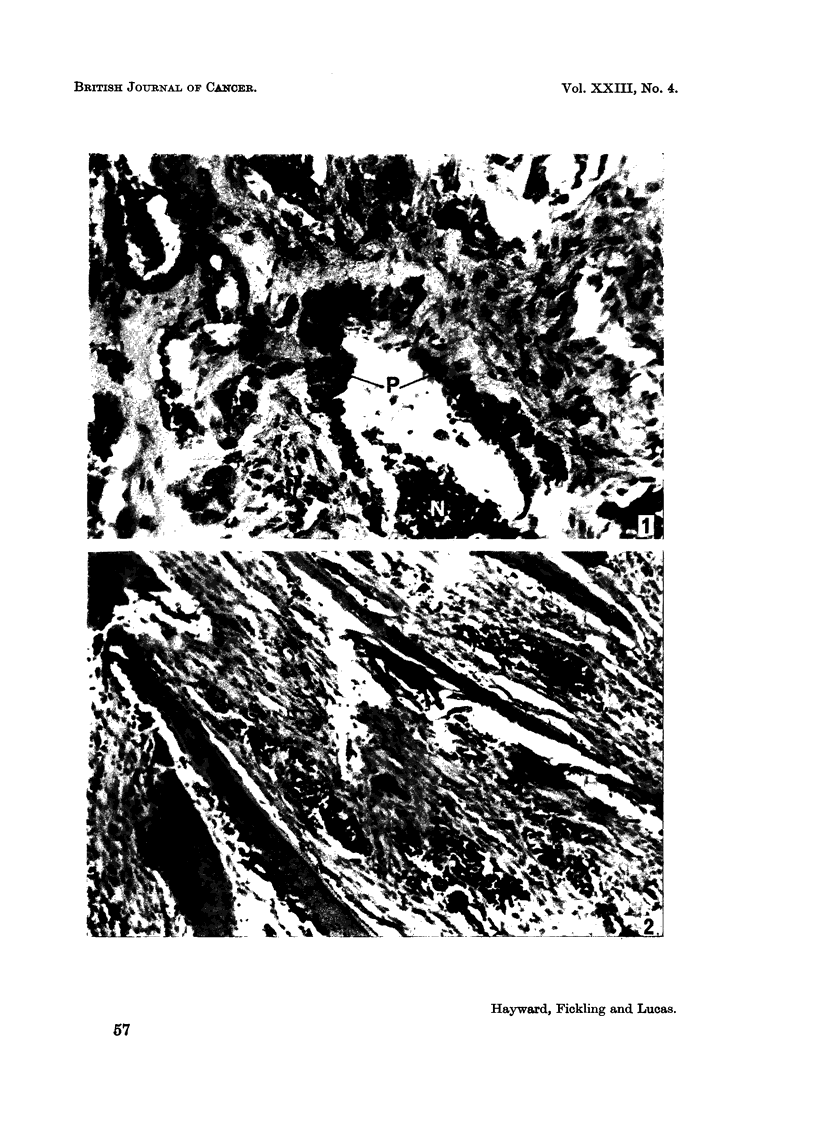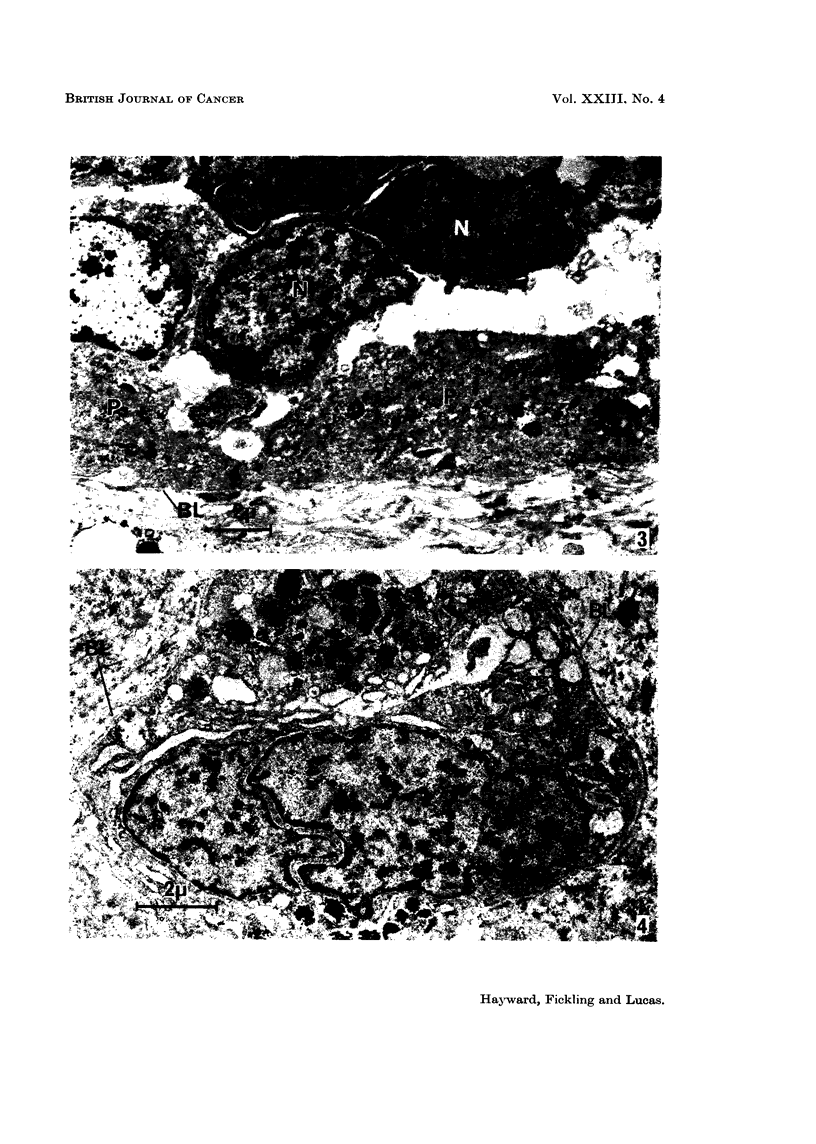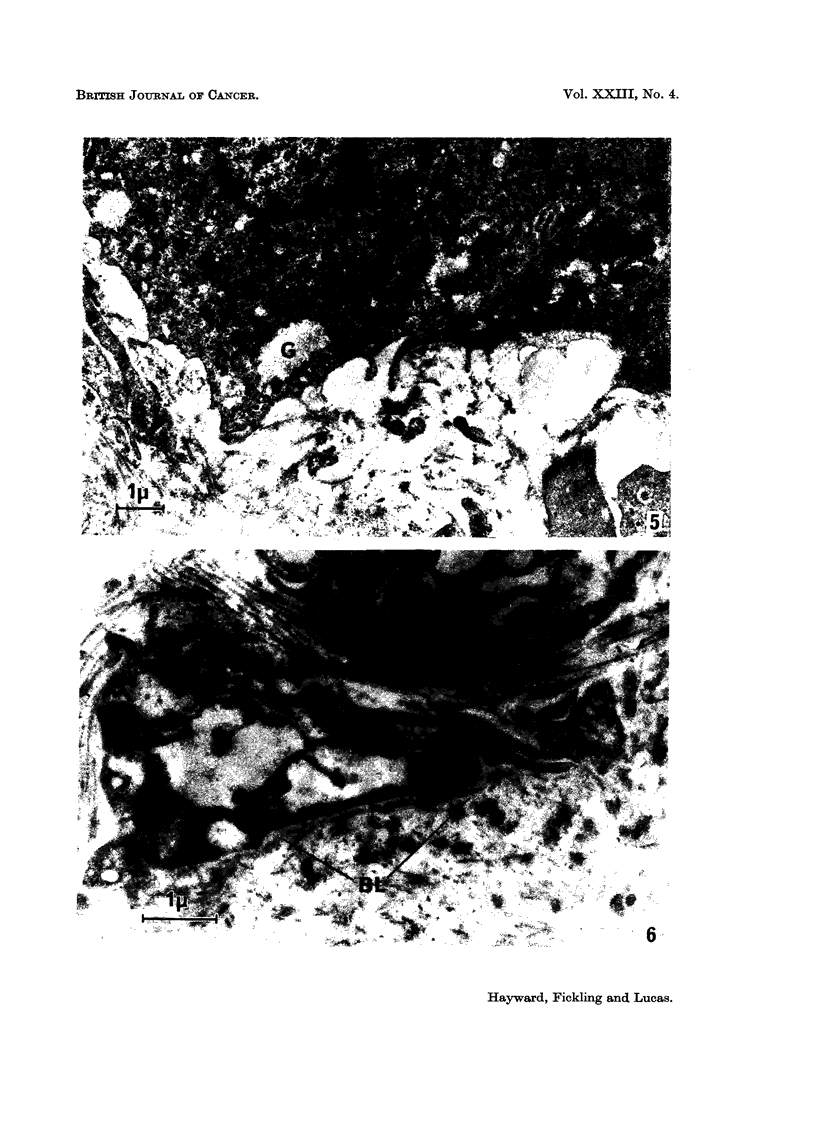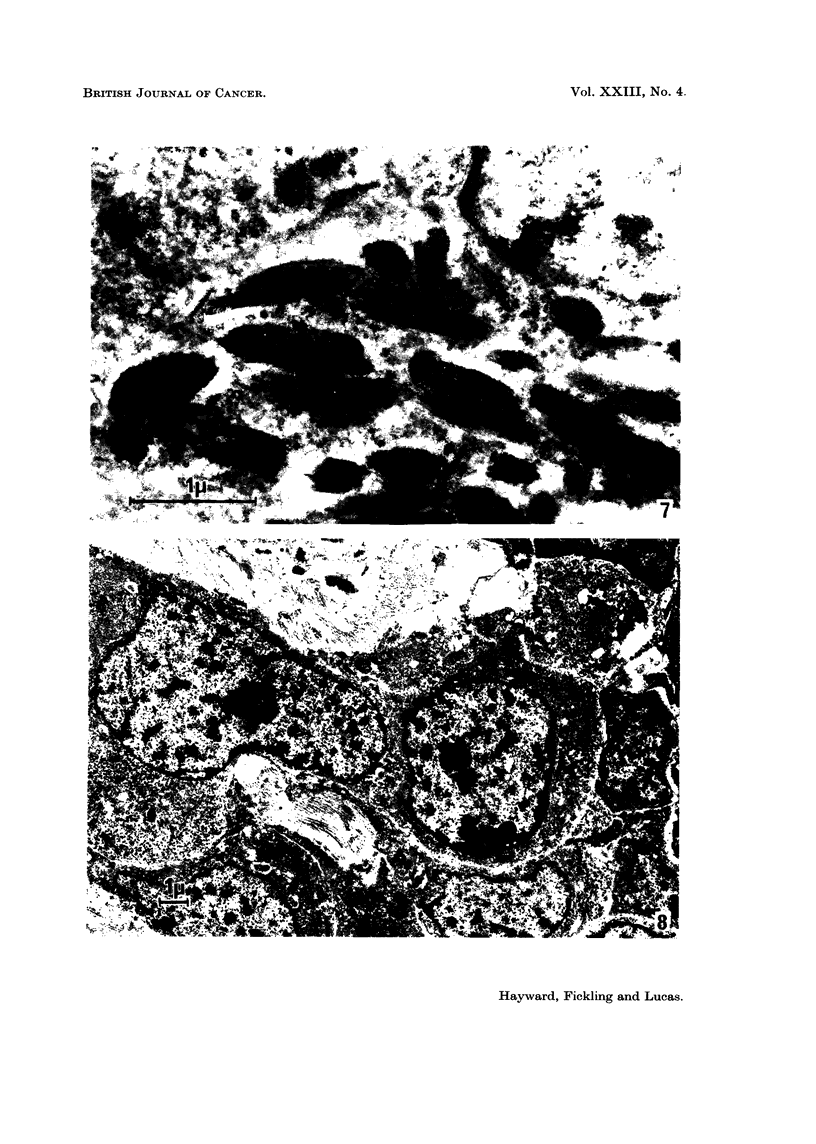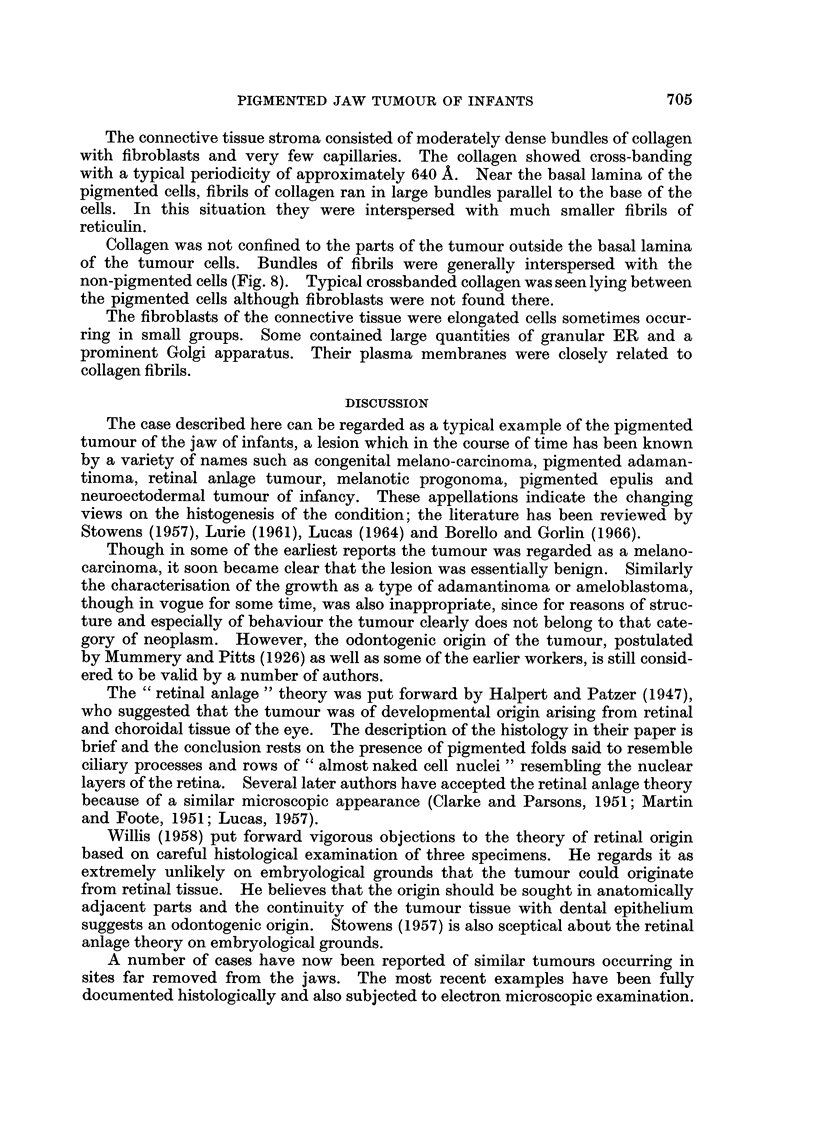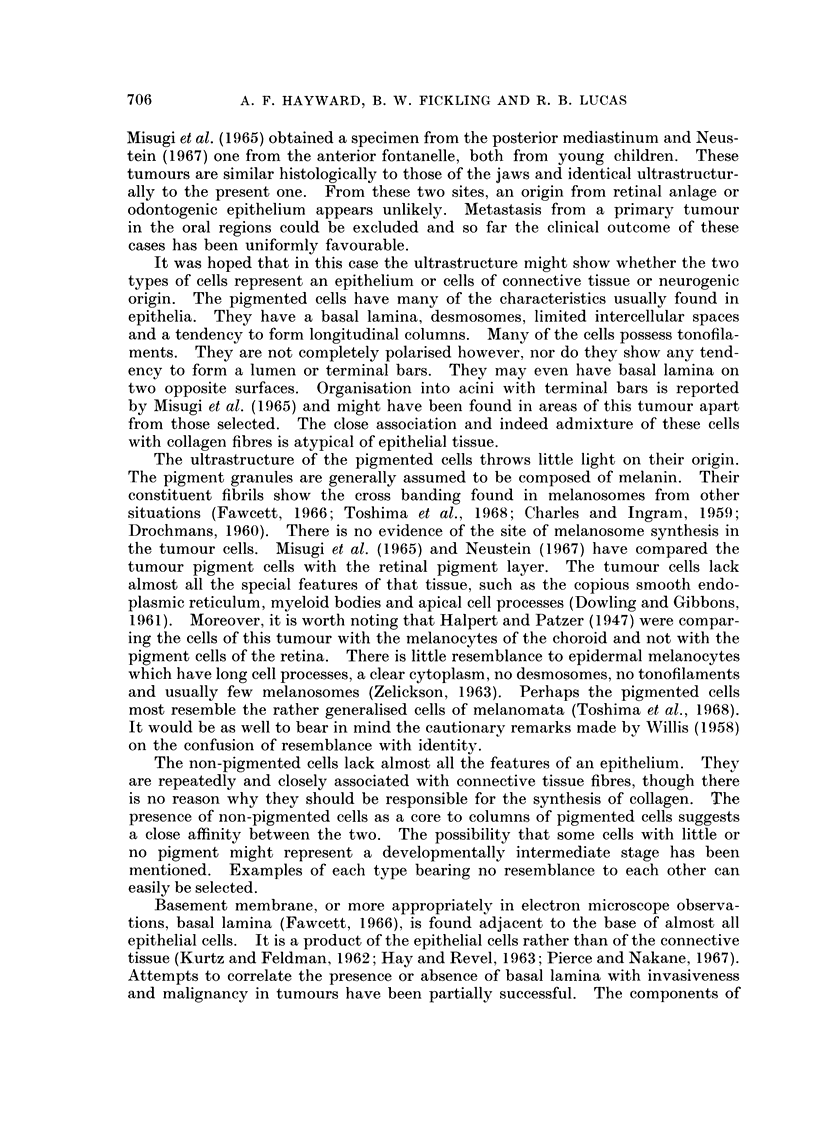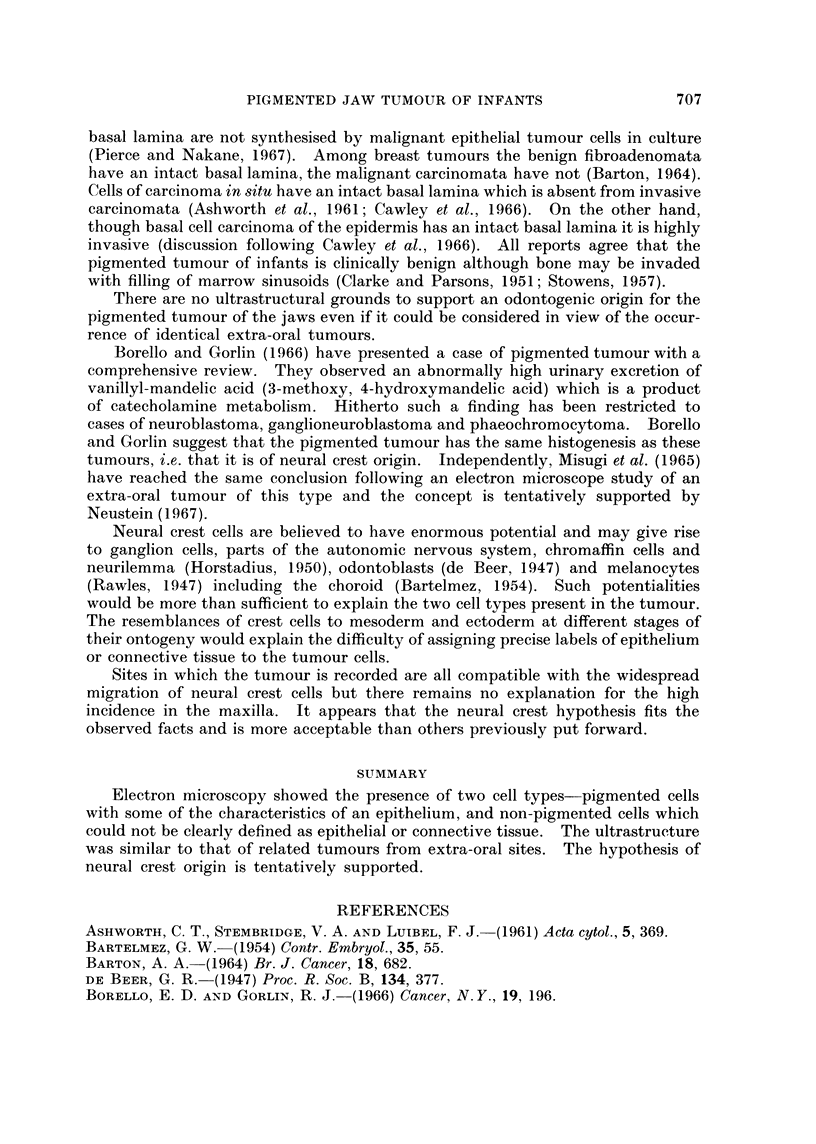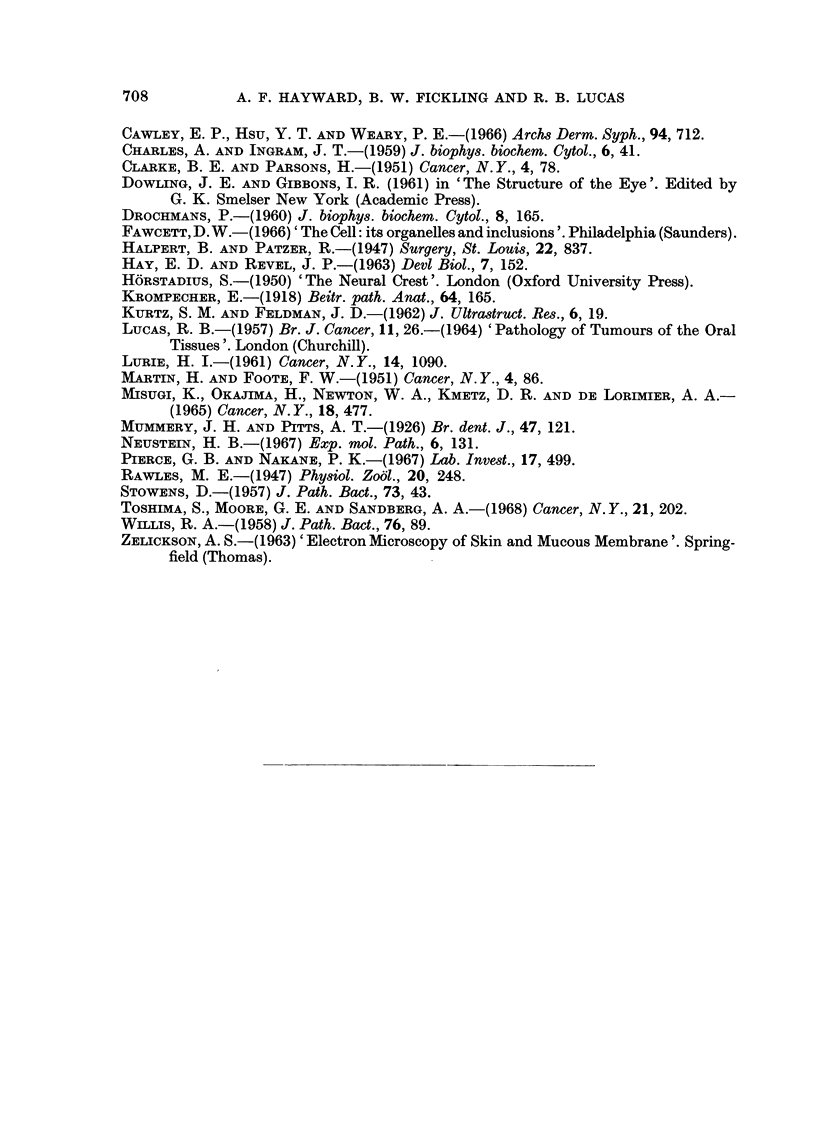# An electron microscope study of a pigmented tumour of the jaw of infants.

**DOI:** 10.1038/bjc.1969.86

**Published:** 1969-12

**Authors:** A. F. Hayward, B. W. Fickling, R. B. Lucas

## Abstract

**Images:**


### AN ELECTRON MICROSCOPE STUDY OF A PIGMENTED TUMOUR OF THE JAW OF INFANTS

A. F. HAYWARD,* B. W. FICKLING† AND R. B. LUCAS‡

*From the departments of Oral Anatomy* and Pathology,‡ Royal Dental Hospital ofLondon School of Dental Surgery, Leicester Square, London, W.C.2, and the Department of Oral and Dental Surgery,† Mount Vernon Hospital, Northwood, Middlesex*

Received for publication August 21, 1969

THE pigmented tumour of the jaw of infants, though rare, is well known to oral pathologists. Probably, it was first described by Krompecher (1918), who reported it as a congenital melanocarcinoma. Since then there has been considerable debate on the histogenesis of the lesion.

Typically, the tumour arises in the jaws, especially the maxilla, of infants under 12 months of age. There have also been reports of tumours of similar histology arising in other parts of the body. Though two such specimens, one from the anterior fontanelle and the other from the mediastinum have been studied by electron microscopy (Misugi *et al.*, 1965; Neustein, 1967), no details of the electron microscopy of oral tumours have been published. It has seemed important, therefore, to confirm the reported similarity between the extra-oral tumours and those of the jaws by examining the ultrastructure of a typical oral example. The results are reported here.

### *Clinical features*

The patient was a female infant aged 7 weeks, with a swelling of the maxilla displacing the right nostril and deforming the buccal sulcus. The swelling appeared to be painless and the child was otherwise normal. Radiographs showed displacement of the right upper central incisor tooth by a mass of basically soft tissue density.

At operation, the tumour was found to be bluish and solid. A plane of cleavage was not readily found, particularly in the palate, and there were extensions of tumour backwards into the palate and upwards to the floor of the nose. The main tumour was enucleated and the extensions were then dissected out, so that the whole area finally appeared to be clear of growth.

The tumour consisted of an ovoid mass of tissue measuring 2 × 1·5 cm., partly covered by oral mucosa. A tooth was attached to the surface. The cut surface showed large areas of bluish-black pigmentation in a grey-white background.

### *Light microscopy*

Microscopic examination showed the tumour to consist of groups of epithelial-like cells in a plentiful connective tissue stroma. The cells had large pale nuclei and were both pigmented and non-pigmented. The pigment was present in the form of elongated granules often aggregated in large masses obscuring all cellular detail. The pigmented cells formed small masses in the stroma or lined small cleft-like spaces. The non-pigmented cells were small and round with a well-stained nucleus and showed occasional mitoses. They occurred in groups, often within the spaces lined by the pigmented cells (Fig. 1).

Periodic acid-Schiff staining together with diastase-treated controls showed that glycogen was widespread though uneven in distribution. It was practically restricted to the pigmented cells. Occasionally a pigmented cell was almost filled with glycogen deposit.

The growing edge of the tumour was not clear cut and no capsule was present, groups of tumour cells appearing to infiltrate the surrounding bone (Fig. 2). No continuity was detected between the tumour cells and the tissue surrounding the adherent tooth but serial sections were not examined.

*Electron microscopy*

All the available material had been placed in 4 per cent formol-saline solution at room temperature immediately after resection. The following day small pieces were removed and cut into 1 mm. cubes, washed in water and post-fixed in buffered 1 per cent osmium tetroxide solution. They were then dehydrated and embedded in Araldite. Thin sections of typical areas were stained with uranyl acetate in methanol and examined with the AEI EM6 electron microscope.

The method of fixation was far from ideal for the observation of ultrastructure. There were areas of very poor fixation though in other places preservation was good. As far as possible the descriptions are based on cells which appeared well fixed. The criteria by which fixation was judged included the presence of an intact plasma membrane and the absence of "extracted" nucleoplasm, swollen endoplasmic reticulum, swollen mitochondria or vacuolated cytoplasm. Mitochondria generally were more translucent than could be regarded as normal. The most heavily pigmented cells were the worst preserved.

Pigmented and non-pigmented cells were easily recognised. Both types occurred in isolation surrounded by connective tissue or in association with each other. The non-pigmented cells were often surrounded by a layer of pigmented cells, sometimes only one cell thick (Fig. 3).

In most places the pigmented cells had the appearance of an epithelium (Fig. 4). They were close together with complex intercellular spaces into which masses of microvillous processes projected. Some of the cells were joined by desmosomes but terminal bars and a differentiated apical surface were not found. At the interface between pigmented cells and connective tissue there was a basal lamina which closely followed the surface of the pigmented cells, crossing the intercellular spaces. It was often discontinuous with substantial gaps (Fig. 5). Collagen was found in the layer of pigmented cells even where the basal lamina appeared to be intact (Fig. 6). Where pigmented cells were arranged in a column only one cell wide, the basal lamina was found on opposite surfaces of the same cell (Fig. 4).

The pigmented cells were characterised by the presence of pigment granules. The cytoplasm was rather complex and varied considerably, especially in its content of granular endoplasmic reticulum (ER). Those cells with the greatest number of granules were largely filled with granular ER which usually showed gross dilatation of its cisternae (Fig. 4). This appearance was believed to be a fixation artefact, to which pigmented cells were particularly prone. Other pigmented cells with very few granules contained a good deal less ER and their bulky cytoplasm was largely filled with free ribosomes and some tonofilaments.

The pigment granules were very electron dense and were either round or spindle-shaped, perhaps according to their plane of section (Fig. 7). They were membrane-bound and in favourable examples were seen to contain bundles of parallel electron dense fibrils. When cut along their long axes, or more especially tangentially, transverse striations or granules were visible in the fibrils. The Golgi apparatus of the pigmented cells was generally inconspicuous but tended to be larger in those cells with most granules. Stages of development of pigment granules like those reported by Neustein (1967) in a similar tumour were not observed.

The non-pigmented cells were close packed in masses, either as the centre of a pigmented column (Fig. 3) or separately in the connective tissue stroma (Fig. 8). They were not surrounded by a basal lamina but there was usually a clear space around each group of cells. Quantities of collagen were found closely associated with the non-pigmented cells (Fig. 8) and it was present even when they were surrounded by pigmented cells with an intact basal lamina. The cells had no desmosomes between them; their outline was smooth with no cell projections. The main cellular characteristic was the small amount of cytoplasm in comparison with the size of the nucleus. In the nucleus, the chromatin was often distributed peripherally. The cytoplasm was packed with free ribosomes and polysomes with a few strands of granular ER.

In their most characteristic forms the two cell types were quite different. Apart from the absence of pigment granules, the non-pigmented cells were recognisable by the comparatively small amount of cytoplasm and poorly developed endoplasmic reticulum. However some cells, also without pigment granules and in close association with typical non-pigmented cells, possessed a greater quantity of cytoplasm and a well developed granular ER. The possibility that these represented stages of development intermediate between non-pigmented and pigmented cells could not be excluded.

---

### EXPLANATION OF PLATES.

Fig. 1.—Light micrograph of pigmented tumour of the jaw showing pigmented cells (P) lining clefts within which lie islands of non-pigmented cells (N). × 100

Fig. 2.—Light micrograph showing infiltration of the boney maxilla with tumour tissue. × 200.

Fig. 3.—Electron micrograph showing a single layer of pigmented cells (P) with sparse pigment granules lying on the basal lamina (BL). The non-pigmented cells (N) with sparse cytoplasm lie in a group separated by a broad space from the pigmented cells. × 5500.

Fig. 4.—Pigmented cells forming a column covered on both sides with basal lamina (BL) surrounded by connective tissue. The cytoplasm shows substantial fixation artefact. × 8500.

Fig. 5.—Tumour cells lying in collagenous connective tissue matrix without an intervening basal lamina. The cell on the right contains pigment granules and organised endoplasmic reticulum. The other contains no pigment granules but contains glycogen (G) and is probably a pigmented cell. × 10,000.

Fig. 6.—The basal parts of two pigmented cells lying on the basal lamina (BL). Collagen can be seen, apparently between the cells and separated from the remaining connective tissue by the basement lamina. In an isolated field such as this the appearance could be due to the plane of sectioning but the juxtaposition of collagen and basal lamina was a regular feature of this part of the tumour. × 16,000.

Fig. 7.—A group of pigment granules from a typical pigmented cell. × 27,000.

Fig. 8.—Non-pigmented cells showing the prominent nucleus and largely ribosomal cytoplasm. These cells are freely interspersed with collagen. × 6000.

Hayward, Fickling and Lucas.

Hayward, Fickling and Lucas.

G
1μ
5

BL
1μ
6

Hayward, Fickling and Lucas.

The connective tissue stroma consisted of moderately dense bundles of collagen with fibroblasts and very few capillaries. The collagen showed cross-banding with a typical periodicity of approximately 640 Å. Near the basal lamina of the pigmented cells, fibrils of collagen ran in large bundles parallel to the base of the cells. In this situation they were interspersed with much smaller fibrils of reticulin.

Collagen was not confined to the parts of the tumour outside the basal lamina of the tumour cells. Bundles of fibrils were generally interspersed with the non-pigmented cells (Fig. 8). Typical crossbanded collagen was seen lying between the pigmented cells although fibroblasts were not found there.

The fibroblasts of the connective tissue were elongated cells sometimes occurring in small groups. Some contained large quantities of granular ER and a prominent Golgi apparatus. Their plasma membranes were closely related to collagen fibrils.

### DISCUSSION

The case described here can be regarded as a typical example of the pigmented tumour of the jaw of infants, a lesion which in the course of time has been known by a variety of names such as congenital melano-carcinoma, pigmented adamantinoma, retinal anlage tumour, melanotic progonoma, pigmented epulis and neuroectodermal tumour of infancy. These appellations indicate the changing views on the histogenesis of the condition; the literature has been reviewed by Stowens (1957), Lurie (1961), Lucas (1964) and Borello and Gorlin (1966).

Though in some of the earliest reports the tumour was regarded as a melano-carcinoma, it soon became clear that the lesion was essentially benign. Similarly the characterisation of the growth as a type of adamantinoma or ameloblastoma, though in vogue for some time, was also inappropriate, since for reasons of structure and especially of behaviour the tumour clearly does not belong to that category of neoplasm. However, the odontogenic origin of the tumour, postulated by Mummery and Pitts (1926) as well as some of the earlier workers, is still considered to be valid by a number of authors.

The " retinal anlage " theory was put forward by Halpert and Patzer (1947), who suggested that the tumour was of developmental origin arising from retinal and choroidal tissue of the eye. The description of the histology in their paper is brief and the conclusion rests on the presence of pigmented folds said to resemble ciliary processes and rows of " almost naked cell nuclei " resembling the nuclear layers of the retina. Several later authors have accepted the retinal anlage theory because of a similar microscopic appearance (Clarke and Parsons, 1951; Martin and Foote, 1951; Lucas, 1957).

Willis (1958) put forward vigorous objections to the theory of retinal origin based on careful histological examination of three specimens. He regards it as extremely unlikely on embryological grounds that the tumour could originate from retinal tissue. He believes that the origin should be sought in anatomically adjacent parts and the continuity of the tumour tissue with dental epithelium suggests an odontogenic origin. Stowens (1957) is also sceptical about the retinal anlage theory on embryological grounds.

A number of cases have now been reported of similar tumours occurring in sites far removed from the jaws. The most recent examples have been fully documented histologically and also subjected to electron microscopic examination.

Misugi *et al.* (1965) obtained a specimen from the posterior mediastinum and Neustein (1967) one from the anterior fontanelle, both from young children. These tumours are similar histologically to those of the jaws and identical ultrastructurally to the present one. From these two sites, an origin from retinal anlage or odontogenic epithelium appears unlikely. Metastasis from a primary tumour in the oral regions could be excluded and so far the clinical outcome of these cases has been uniformly favourable.

It was hoped that in this case the ultrastructure might show whether the two types of cells represent an epithelium or cells of connective tissue or neurogenic origin. The pigmented cells have many of the characteristics usually found in epithelia. They have a basal lamina, desmosomes, limited intercellular spaces and a tendency to form longitudinal columns. Many of the cells possess tonofilaments. They are not completely polarised however, nor do they show any tendency to form a lumen or terminal bars. They may even have basal lamina on two opposite surfaces. Organisation into acini with terminal bars is reported by Misugi *et al.* (1965) and might have been found in areas of this tumour apart from those selected. The close association and indeed admixture of these cells with collagen fibres is atypical of epithelial tissue.

The ultrastructure of the pigmented cells throws little light on their origin. The pigment granules are generally assumed to be composed of melanin. Their constituent fibrils show the cross banding found in melanosomes from other situations (Fawcett, 1966; Toshima *et al.*, 1968; Charles and Ingram, 1959; Drochmans, 1960). There is no evidence of the site of melanosome synthesis in the tumour cells. Misugi *et al.* (1965) and Neustein (1967) have compared the tumour pigment cells with the retinal pigment layer. The tumour cells lack almost all the special features of that tissue, such as the copious smooth endoplasmic reticulum, myeloid bodies and apical cell processes (Dowling and Gibbons, 1961). Moreover, it is worth noting that Halpert and Patzer (1947) were comparing the cells of this tumour with the melanocytes of the choroid and not with the pigment cells of the retina. There is little resemblance to epidermal melanocytes which have long cell processes, a clear cytoplasm, no desmosomes, no tonofilaments and usually few melanosomes (Zelickson, 1963). Perhaps the pigmented cells most resemble the rather generalised cells of melanomata (Toshima *et al.*, 1968). It would be as well to bear in mind the cautionary remarks made by Willis (1958) on the confusion of resemblance with identity.

The non-pigmented cells lack almost all the features of an epithelium. They are repeatedly and closely associated with connective tissue fibres, though there is no reason why they should be responsible for the synthesis of collagen. The presence of non-pigmented cells as a core to columns of pigmented cells suggests a close affinity between the two. The possibility that some cells with little or no pigment might represent a developmentally intermediate stage has been mentioned. Examples of each type bearing no resemblance to each other can easily be selected.

Basement membrane, or more appropriately in electron microscope observations, basal lamina (Fawcett, 1966), is found adjacent to the base of almost all epithelial cells. It is a product of the epithelial cells rather than of the connective tissue (Kurtz and Feldman, 1962; Hay and Revel, 1963; Pierce and Nakane, 1967). Attempts to correlate the presence or absence of basal lamina with invasiveness and malignancy in tumours have been partially successful. The components of basal lamina are not synthesised by malignant epithelial tumour cells in culture (Pierce and Nakane, 1967). Among breast tumours the benign fibroadenomata have an intact basal lamina, the malignant carcinomata have not (Barton, 1964). Cells of carcinoma *in situ* have an intact basal lamina which is absent from invasive carcinomata (Ashworth *et al.*, 1961; Cawley *et al.*, 1966). On the other hand, though basal cell carcinoma of the epidermis has an intact basal lamina it is highly invasive (discussion following Cawley *et al.*, 1966). All reports agree that the pigmented tumour of infants is clinically benign although bone may be invaded with filling of marrow sinusoids (Clarke and Parsons, 1951; Stowens, 1957).

There are no ultrastructural grounds to support an odontogenic origin for the pigmented tumour of the jaws even if it could be considered in view of the occurrence of identical extra-oral tumours.

Borello and Gorlin (1966) have presented a case of pigmented tumour with a comprehensive review. They observed an abnormally high urinary excretion of vanillyl-mandelic acid (3-methoxy, 4-hydroxymandelic acid) which is a product of catecholamine metabolism. Hitherto such a finding has been restricted to cases of neuroblastoma, ganglioneuroblastoma and phaeochromocytoma. Borello and Gorlin suggest that the pigmented tumour has the same histogenesis as these tumours, *i.e.* that it is of neural crest origin. Independently, Misugi *et al.* (1965) have reached the same conclusion following an electron microscope study of an extra-oral tumour of this type and the concept is tentatively supported by Neustein (1967).

Neural crest cells are believed to have enormous potential and may give rise to ganglion cells, parts of the autonomic nervous system, chromaffin cells and neurilemma (Horstadius, 1950), odontoblasts (de Beer, 1947) and melanocytes (Rawles, 1947) including the choroid (Bartelmez, 1954). Such potentialities would be more than sufficient to explain the two cell types present in the tumour. The resemblances of crest cells to mesoderm and ectoderm at different stages of their ontogeny would explain the difficulty of assigning precise labels of epithelium or connective tissue to the tumour cells.

Sites in which the tumour is recorded are all compatible with the widespread migration of neural crest cells but there remains no explanation for the high incidence in the maxilla. It appears that the neural crest hypothesis fits the observed facts and is more acceptable than others previously put forward.

### SUMMARY

Electron microscopy showed the presence of two cell types—pigmented cells with some of the characteristics of an epithelium, and non-pigmented cells which could not be clearly defined as epithelial or connective tissue. The ultrastructure was similar to that of related tumours from extra-oral sites. The hypothesis of neural crest origin is tentatively supported.

### REFERENCES

ASHWORTH, C. T., STEMBRIDGE, V. A. AND LUIBEL, F. J.—(1961) *Acta cytol.*, **5**, 369.
BARTELMEZ, G. W.—(1954) *Contr. Embryol.*, **35**, 55.
BARTON, A. A.—(1964) *Br. J. Cancer*, **18**, 682.
DE BEER, G. R.—(1947) *Proc. R. Soc.* B, **134**, 377.
BORELLO, E. D. AND GORLIN, R. J.—(1966) *Cancer, N.Y.*, **19**, 196.

Cawley, E. P., Hsu, Y. T. and Weary, P. E.—(1966) *Archs Derm. Syph.*, **94**, 712.
Charles, A. and Ingram, J. T.—(1959) *J. biophys. biochem. Cytol.*, **6**, 41.
Clarke, B. E. and Parsons, H.—(1951) *Cancer, N.Y.*, **4**, 78.
Dowling, J. E. and Gibbons, I. R. (1961) in 'The Structure of the Eye'. Edited by G. K. Smelser New York (Academic Press).
Drochmans, P.—(1960) *J. biophys. biochem. Cytol.*, **8**, 165.
Fawcett, D. W.—(1966) 'The Cell: its organelles and inclusions'. Philadelphia (Saunders).
Halpert, B. and Patzer, R.—(1947) *Surgery, St. Louis*, **22**, 837.
Hay, E. D. and Revel, J. P.—(1963) *Devl Biol.*, **7**, 152.
Hörstadius, S.—(1950) 'The Neural Crest'. London (Oxford University Press).
Krompecher, E.—(1918) *Beitr. path. Anat.*, **64**, 165.
Kurtz, S. M. and Feldman, J. D.—(1962) *J. Ultrastruct. Res.*, **6**, 19.
Lucas, R. B.—(1957) *Br. J. Cancer*, **11**, 26.—(1964) 'Pathology of Tumours of the Oral Tissues'. London (Churchill).
Lurie, H. I.—(1961) *Cancer, N.Y.*, **14**, 1090.
Martin, H. and Foote, F. W.—(1951) *Cancer, N.Y.*, **4**, 86.
Misugi, K., Okajima, H., Newton, W. A., Kmetz, D. R. and de Lorimier, A. A.—(1965) *Cancer, N.Y.*, **18**, 477.
Mummery, J. H. and Pitts, A. T.—(1926) *Br. dent. J.*, **47**, 121.
Neustein, H. B.—(1967) *Exp. mol. Path.*, **6**, 131.
Pierce, G. B. and Nakane, P. K.—(1967) *Lab. Invest.*, **17**, 499.
Rawles, M. E.—(1947) *Physiol. Zoöl.*, **20**, 248.
Stowens, D.—(1957) *J. Path. Bact.*, **73**, 43.
Toshima, S., Moore, G. E. and Sandberg, A. A.—(1968) *Cancer, N.Y.*, **21**, 202.
Willis, R. A.—(1958) *J. Path. Bact.*, **76**, 89.
Zelickson, A. S.—(1963) 'Electron Microscopy of Skin and Mucous Membrane'. Springfield (Thomas).